# Machine Learning-Based Prediction of No-Show Telemedicine Encounters

**DOI:** 10.1089/tmr.2025.0009

**Published:** 2025-04-07

**Authors:** C. Mahony Reategui-Rivera, Wanting Cui, Stefan Escobar-Agreda, Leonardo Rojas-Mezarina, Joseph Finkelstein

**Affiliations:** ^1^ Department of Biomedical Informatics, School of Medicine, University of Utah, Salt Lake City, Utah, USA.; ^2^ Telehealth Unit, School of Medicine, Universidad Nacional Mayor de San Marcos, Lima, Peru.

**Keywords:** artificial intelligence, machine learning, no-show, Peru, prediction, telehealth, telemedicine

## Abstract

**Aim:**

This study aimed to evaluate the performance of machine learning (ML) models in predicting patient no-shows for telemedicine appointments within Peruvian health system and identify key predictors of nonattendance.

**Methods:**

We performed a retrospective observational study using anonymized data (June 2019–November 2023) from “Teleatiendo.” The dataset included over 1.5 million completed appointments and about 64,000 no-shows (4.1%), focusing on teleorientation and telemonitoring. Predictor variables included patient demographics, socioeconomic factors, health care facility characteristics, appointment timing, and telemedicine service types. A 70% training, 10% validation, and 20% testing split were used over 10 iterations, with hyperparameter tuning performed on the validation set to identify optimal model parameters. Multiple ML approaches—random forest, XGBoost, LightGBM, and anomaly detection—were implemented in combination with undersampling and cost-sensitive learning to address class imbalance. Performance was evaluated using precision, recall, specificity, area under the curve (AUC), F1-score, and accuracy.

**Results:**

Of the models tested, undersampling with XGBoost achieved a precision of 0.115 (±0.001), recall of 0.654 (±0.005), specificity of 0.786 (±0.002), AUC of 0.720 (±0.002), and accuracy of 0.780 (±0.002). In contrast, cost-sensitive XGBoost exhibited a balanced performance with a precision of 0.123 (±0.001), recall of 0.639 (±0.006), specificity of 0.805 (±0.004), AUC of 0.722 (±0.001), and accuracy of 0.799 (±0.003). Additionally, cost-sensitive random forest achieved the highest specificity (0.843 ± 0.002) and accuracy (0.832 ± 0.001) but recorded a lower recall (0.585 ± 0.004), while cost-sensitive LightGBM and balanced random forest yielded performance metrics similar to cost-sensitive XGBoost. Isolation forest, used for abnormality detection, demonstrated the lowest performance.

**Conclusions:**

ML models can moderately predict telemedicine no-shows in Peru, with cost-sensitive boosting techniques enhancing the identification of high-risk patients. Key predictors reflect both individual behavior and system-level contexts, suggesting the need for tailored, context-specific interventions. These findings can inform targeted strategies to optimize telemedicine, improve appointment adherence, and promote equitable health care access.

## Introduction 

### Impact of no-shows on health care

Patient no-shows at medical appointments pose a significant challenge to health care systems globally, affecting service efficiency and patient welfare.^1^ In Peru, where resources are limited, about 20% of patients fail to attend scheduled appointments, leading to resource underutilization and disrupted care continuity.^2,3^ This underutilization strains health care budgets and delays care for other patients, exacerbating access issues in already stretched systems.

### No-shows in telemedicine

The advent of telemedicine has introduced new dynamics in health care service provision and appointment management, with patient no-show behavior potentially differing from that in outpatient settings.^4–6^ Factors such as medical specialty, care mode, and geographical region contribute to the variability in patient profiles.

### Artificial intelligence prediction of no-shows

While traditional approaches like reminder systems and patient education have been employed to mitigate no-show rates, they are often constrained by costs, logistical complexities, and potential ethical concerns.^7,8^ In contrast, machine learning (ML) offers a promising alternative that can analyze vast datasets to identify patterns and predict high-risk no-show patients.^9^ This predictive capability enables health care providers to implement targeted and cost-effective interventions. While studies in the United States have demonstrated the high performance of ML models in predicting no-show rates in telemedicine,^10^ there is a paucity of research on their performance in diverse international contexts, particularly in developing countries like Peru.

Given this backdrop, our study aims to bridge this knowledge gap by evaluating the performance of ML models to predict no-shows in telemedicine encounters within a comprehensive telehealth information system in Peru.

## Methods

### Study design

We conducted a retrospective observational study utilizing secondary data from the health information system “Teleatiendo,” covering the period from June 2020 to November 2023. The methodology and reporting adhere to the Guidelines for Developing and Reporting Machine Learning Predictive Models in Biomedical Research.^11^

### Data source

“Teleatiendo” is a web-based health information system managed by the Peruvian Ministry of Health. It facilitates the scheduling and registration of telemedicine encounters from both public health facilities across Peru. The system captures administrative data (e.g., appointment dates and times, assignments, health facility characteristics) and clinical data (e.g., patient demographics, encounter types).

Telemedicine services offered through Teleatiendo are regulated by the Peruvian Telehealth Law,^12,13^ which classifies these services into four main types: (i) Teleconsultation, enables remote patient care, including diagnosis and treatment; (ii) teleinterconsultation, allows health care professionals to consult each other about a patient’s case; (iii) teleorientation, provides remote guidance on health promotion and disease prevention; and (iv) telemonitoring, involves continuous remote tracking of a patient’s health parameters. Table 1 provides a detailed description of each category to contextualize the range of telemedicine services included in the system.

**Table 1.** Types of Telemedicine According to Peruvian Telehealth Law

Telemedicine type	Description
Teleconsultation	A remote consultation between a health professional within their scope of expertise and a patient using ICT (Information and Communication Technologies). The purpose of teleconsultation is to provide services related to health promotion, prevention, diagnosis, treatment, recovery, rehabilitation, and palliative care, as applicable. It adheres to regulated restrictions on prescription medications and other provisions determined by the Ministry of Health.
Teleinterconsultation	This is a remote consultation conducted via ICT by one health professional to another for the care of a patient who may or may not be present. The aims include health promotion, prevention, diagnosis, treatment, recovery, rehabilitation, and palliative care following regulated prescription restrictions and other determinations by the Ministry of Health.
Teleorientation	This involves activities conducted by a health professional using ICT to provide health users with counseling and advice for health promotion, disease prevention, recovery, or rehabilitation.
Telemonitoring	This refers to the remote monitoring of a patient in health care institutions, where clinical information and, as medically warranted, biomedical parameters, and/or auxiliary tests are transmitted to manage the patient’s health condition. Medication prescription may or may not be included, depending on medical judgment and the competencies of other health professionals.

**FIG. 1.**
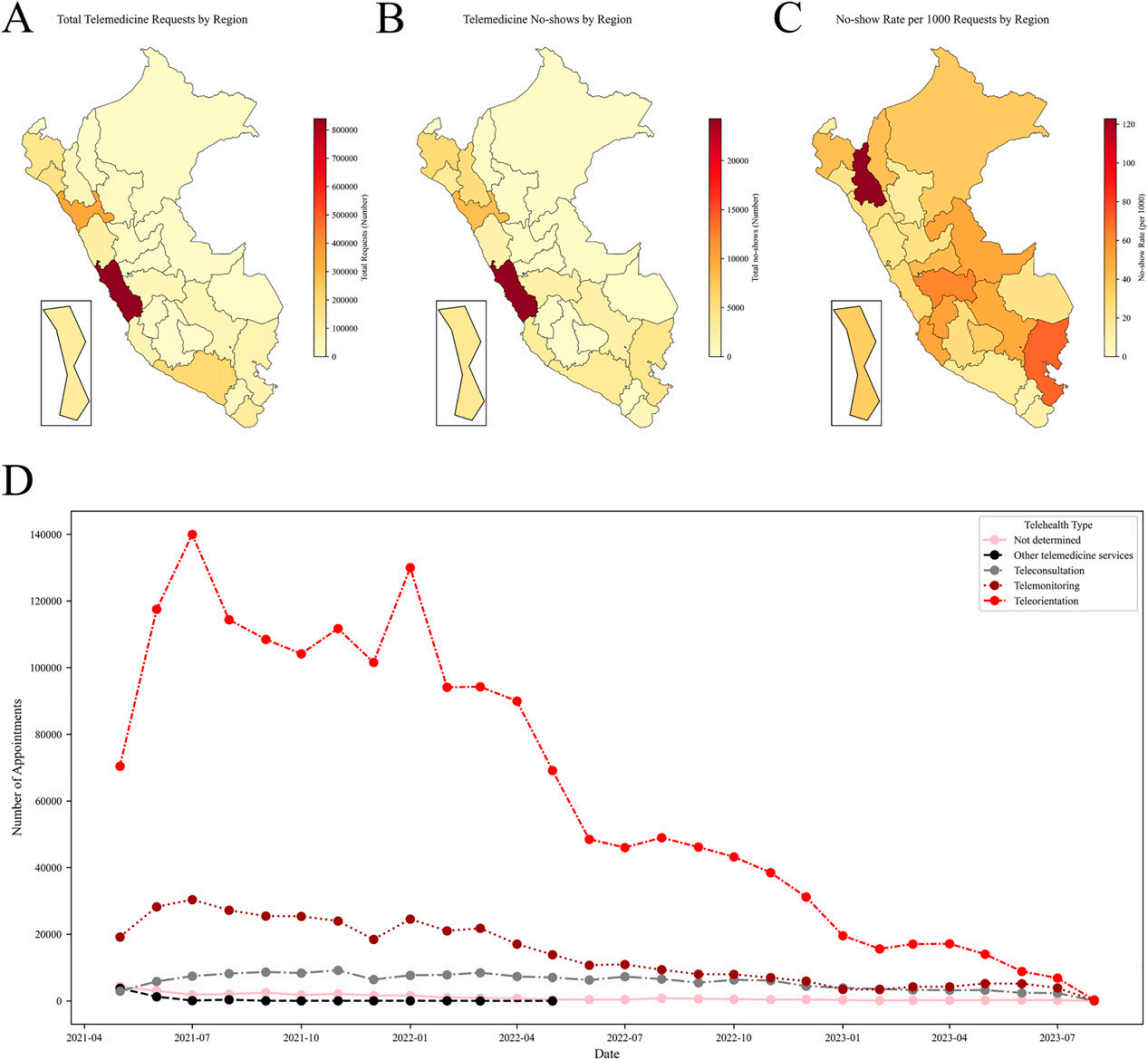
Coverage of Teleatiendo appointments across Peruvian territory. **(A)** Map of appointments requested. **(B)** Map of appointments done. **(C)** Map of no-show rate. **(D)** Trends of use of different types of telemedicine.

For this study, we obtained deidentified data from Teleatiendo through formal requests to the Ministry of Health. The dataset was anonymized using the Shift and Truncation method, which randomly shifts dates within a ±7-day range while preserving temporal relationships.^14^ Figure 1 provides an overview of the geographic coverage of telemedicine appointments in Peru, the distribution of no-shows, and trends in telemedicine usage over time.

### Sample

We included records with complete data on specified features and outcomes, focusing on teleorientation and telemonitoring categories. Records were excluded if they had missing predictor variables, duplicate entries, or intermediate statuses (“to be assigned” or “assigned”). We also excluded patients with more than 10 visits to avoid overrepresentation.

To ensure sufficient stability of the models, we empirically utilized the total available observations. We calculated the required sample size based on the rule of 10 events per variable, adjusted for the no-show rate of 4%. With 15 predictors, we determined a minimum sample size of 7895 appointments.^15^

### Variables

The primary outcome was a binary variable indicating whether a patient attended a scheduled telemedicine appointment (0 = attended, 1 = no-show). Predictor variables included:

• Patient characteristics: age, gender, region of residence, rurality, wealth index, and insurance type.
• Health facility attributes: complexity level, facility wealth index, and rurality.
• Appointment details: schedule hour, assignment hour, and telemedicine type.

Three additional variables were derived based on historical data: the number of previous encounters, percentage of prior no-shows, and days between the most recent previous visit and the current appointment.

No-show rates were compared across different visit characteristics using chi-square tests for categorical variables, *t*-tests for normally distributed numeric variables, and Mann–Whitney tests for non-normally distributed numeric variables.

### Machine learning analysis

To evaluate the performance of predictive models, we used metrics including precision, recall (sensitivity), specificity, receiver operating characteristic–area under the curve (AUC-ROC), and accuracy.^11^ Precision measured the accuracy of positive predictions (no-shows), recall assessed the model’s ability to identify all relevant instances, and specificity evaluated the correct identification of actual negatives (attended cases). The AUC-ROC provided an aggregate performance measure across all classification thresholds and was calculated using the roc_auc_score function from scikit-learn, which applies the trapezoidal rule for estimation. Accuracy reflected the overall correctness of predictions.

Given the imbalanced nature of the dataset, where only 4% of cases were no-shows, we implemented several strategies to address challenges associated with predicting the minority class. Undersampling was used to reduce the number of majority class samples, creating a more balanced dataset that improved the model’s sensitivity to no-shows. Additionally, cost-sensitive learning was applied by assigning higher penalties to misclassified no-show cases during training. This approach was particularly effective for tree-based models, such as XGBoost and LightGBM, by incorporating class weights that allowed the models to focus on minority class predictions without altering the dataset size.

We explored both boosting and bagging techniques for this analysis. Boosting models, including XGBoost and LightGBM, build sequential models that learn iteratively from previous errors, making them highly effective in handling class imbalance. Bagging models, such as random forest, aggregate predictions from multiple decision trees trained on bootstrapped samples, which stabilizes predictions and enhances performance when combined with cost-sensitive learning. Furthermore, we evaluated abnormality detection methods, such as isolation forest, which treat no-shows as anomalies within the dataset. These approaches aim to detect rare events by identifying patterns and behaviors that deviate from the majority class.

The final models we chose to evaluate were XGBoost with undersampling, XGBoost with cost-sensitive learning, random forest with cost-sensitive learning, and LightGBM with cost-sensitive learning, balanced random forest, and isolation forest.

**FIG. 2.**
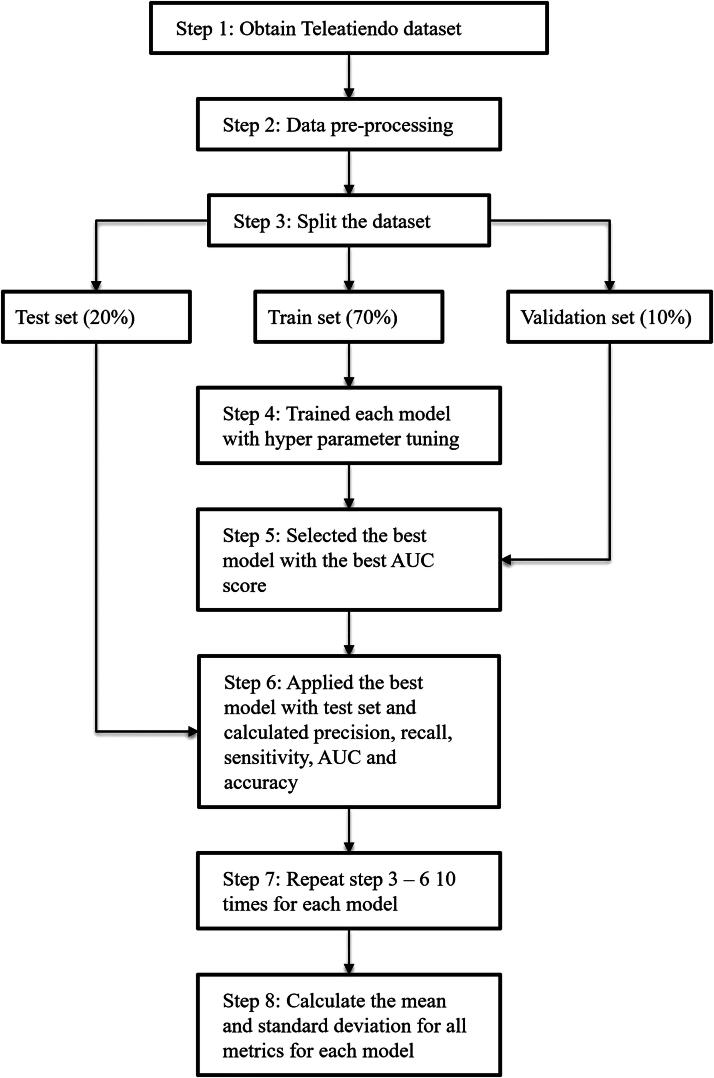
Flowchart of machine learning process.

To identify the best ML model, we performed 10 independent runs for each model. The analytical workflow is depicted in Figure 2. In each iteration, the predictive dataset was randomly split into 70% training, 10% validation, and 20% testing set. The training set was used to build and train the model. We performed hyperparameter tuning by applying different hyperparameters combinations on the training set and evaluating them on the validation set. Since the dataset was imbalanced, we selected the set of parameters with the best AUC score on the validation set. Then, these optimal parameters were applied to the test set to evaluate the performance. For each run, we calculated precision, recall, specificity, AUC, and accuracy scores. To account for the variability in the model performance, we repeated this entire process 10 times and calculated the mean and standard deviation of these metrics.

We verified that the training, validation, and testing sets for each iteration had equivalent distributions of complete versus no-shows using a chi-square test, confirming no significant differences between the sets. All cohorts had a 4% no-show rate.

We used Python 3.10.13 with Jupyter Notebook 4.1.2. ML libraries included scikit-learn 1.3.0, imbalanced-learn 0.11.0, XGBoost 2.0.3, and LightGBM 4.1.0.

## Results

### Descriptive statistics

**FIG. 3.**
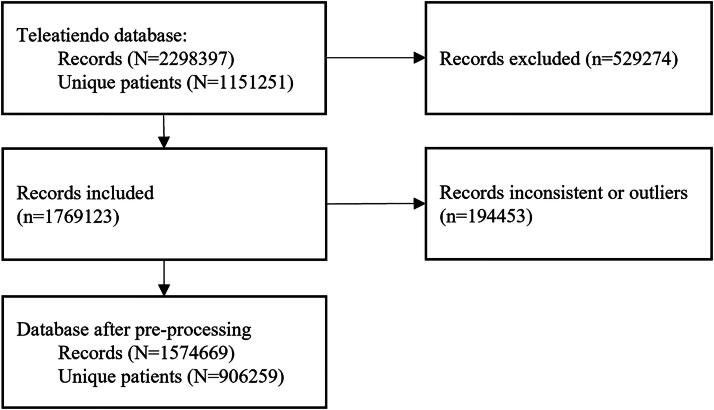
Flowchart of study sample.

The initial dataset included 2,298,397 telemedicine appointments for 1,151,251 patients. After applying the inclusion criteria and preprocessing steps, a final dataset of 1,574,670 records was retained. Of these, 1,510,297 appointments (95.9%) were classified as attended and 64,373 (4.1%) as no-shows (see Fig. 3 for details).

The average age of patients who attended appointments was 36.6 ± 20.9 years, while those classified as no-shows had an average age of 21.7 ± 36.5 years (see Table 2).

**Table 2.** Descriptive Statistics of the Dataset (the Percentages Are Calculated Separately for Each Stratum)

Variables	Attended (*n* = 1,510,297, 95.9%)	No-show (*n* = 64,373, 4.1%)	*p*
** **			
Age	36.6 ± 20.9	21.7 ± 36.5	<0.001
Gender			
Female	1,007,870 (66.73%)	43,070 (66.91%)	0.360
Male	502,427 (33.27%)	21,303 (33.09%)	0.360
Wealth index			
Poorest	30,203 (2.00%)	2472 (3.84%)	<0.001
Poor	44,877 (2.97%)	5140 (7.98%)	<0.001
Middle	62,955 (4.17%)	3470 (5.39%)	<0.001
Rich	330,316 (21.87%)	14,012 (21.77%)	0.532
Richest	1,041,946 (68.99%)	39,279 (61.02%)	<0.001
Rurality			
Rural	156,980 (10.39%)	11,209 (17.41%)	<0.001
Urban	1,353,317 (89.61%)	53,164 (82.59%)	<0.001
Insurance type			
Armed forces and police	4397 (0.29%)	94 (0.15%)	<0.001
Ministry of Health insurance	1,128,767 (74.74%)	53,567 (83.21%)	<0.001
Others	68,106 (4.51%)	2101 (3.26%)	<0.001
Private insurance	4484 (0.30%)	50 (0.08%)	<0.001
Social insurance	304,543 (20.16%)	8561 (13.30%)	<0.001
Health facility			
Complexity level			
Large-sized hospital	267,444 (17.71%)	12,368 (19.21%)	<0.001
Mid-sized hospital	296,500 (19.63%)	26,788 (41.61%)	<0.001
Primary healthcare facility	946,353 (62.66%)	25,217 (39.17%)	<0.001
Wealth index			
Poorest	9773 (0.65%)	143 (0.22%)	<0.001
Poor	27,540 (1.82%)	5467 (8.49%)	<0.001
Middle	51,328 (3.40%)	1659 (2.58%)	<0.001
Rich	334,924 (22.18%)	13,120 (20.38%)	<0.001
Richest	1,086,732 (71.95%)	43,984 (68.33%)	<0.001
Rurality			
Rural	101,121 (6.70%)	6895 (10.71%)	<0.001
Urban	1,409,176 (93.30%)	57,478 (89.29%)	<0.001
Appointment			
Assigning hour			
Afternoon	592,503 (39.23%)	22,341 (34.71%)	<0.001
Evening	408,477 (27.05%)	12,097 (18.79%)	<0.001
Midnight	17,358 (1.15%)	788 (1.22%)	0.082
Morning	491,959 (32.57%)	29,147 (45.28%)	<0.001
Type of telemedicine			
Telemonitoring	211,021 (13.97%)	10,974 (17.05%)	<0.001
Teleorientation	1,299,276 (86.03%)	53,399 (82.95%)	<0.001
Previous encounter	1.0 ± 1.6	1.6 ± 1.0	<0.001
Interval between visits (days)	240.7 ± 156.9	257.4 ± 153.0	<0.001
No-show history (percent)	1.2 ± 9.1	27.9 ± 10.7	<0.001

ML, machine learning.

There was no significant difference in gender between the attended and no-show cohorts, with both consisting of 66% female and 33% male patients. However, there were significant differences in the wealth index. Patients with a low wealth index (poorest and poor) made up >11% of the no-show cohort compared to 5% of the attended cohort. In contrast, patients with the highest wealth index (richest) had significantly higher proportions in the attended cohort (69%) compared to the no-show cohort (61%). Additionally, patients in rural areas had a higher proportion in the no-show cohort (17%) compared to the attended cohort (10%).

Insurance type was also a significant factor. Patients covered by the Ministry of Health insurance had a higher proportion in the no-show cohort (83%) compared to the attended cohort (75%). Patients with social or private insurance had lower proportions in the no-show cohort.

Regarding health care facilities, primary health care facilities had the highest proportion of attended appointments (63%) and a lower proportion of no-shows (39%). In contrast, mid-sized hospitals had a significantly higher proportion of no-shows, with 42% of missed appointments compared to only 20% of attending visits.

Morning appointments had a higher proportion in the no-show cohort (45%) compared to the attended cohort (33%). Additionally, patients who used telemonitoring services had a higher proportion in the no-show cohort (17%) compared to the attended cohort (14%).

Furthermore, no-show patients had a significantly higher history of previously missed appointments (27.9%) compared to those who attended (1.2%).

**FIG. 4.**
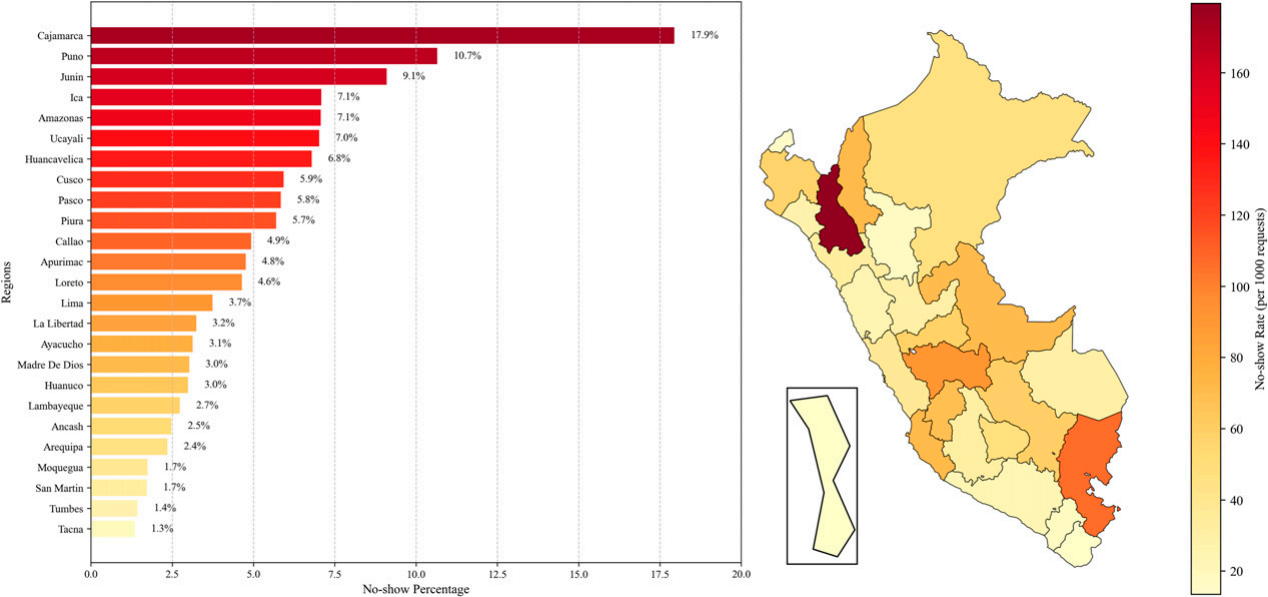
Geographical distribution of no-shows in Peru.

Figure 4 displays the geographical distribution of no-show cases. Cajamarca (17.5%), Puno (10.7%), and Junín (9.1%) had the highest no-show rates, while Tacna (1.3%) and Tumbes (1.4%) exhibited the lowest. In terms of appointment timing, morning appointments had the highest no-show rates (5.6%), compared to evening appointments (2.9%).

### Predictive models’ performance

The performance of the predictive models varied across the different techniques (see Table 3 for details). The XGBoost model with undersampling achieved a precision of 0.115 (±0.001), recall of 0.654 (±0.005), specificity of 0.786 (±0.002), AUC of 0.720 (±0.002), and accuracy of 0.780 (±0.002).

**Table 3.** **Prediction Models Performance in the Test Dataset** ^a^

	Precision	Recall	Specificity	AUC	Accuracy
Undersampling	** **	** **	** **	** **	** **
XGBoost	0.115 (0.001)	0.654 (0.005)	0.786 (0.002)	0.720 (0.002)	0.780 (0.002)
Cost-sensitive learning					
Random forest	0.137 (0.001)	0.585 (0.004)	0.843 (0.002)	0.714 (0.001)	0.832 (0.001)
XGBoost	0.123 (0.001)	0.639 (0.006)	0.805 (0.004)	0.722 (0.001)	0.799 (0.003)
Light GBM	0.123 (0.002)	0.644 (0.006)	0.805 (0.004)	0.724 (0.002)	0.799 (0.004)
Balanced random forest	0.124 (0.001)	0.624 (0.004)	0.813 (0.002)	0.718 (0.001)	0.805 (0.002)
Abnormality detection					
Isolation forest	0.052 (0.002)	0.576 (0.082)	0.545 (0.080)	0.561 (0.003)	0.547 (0.074)

^a^ The cells in the table represent the mean and standard deviation of the performance metrics for each model across 10 iterations.

AUC, area under curve.

Among the cost-sensitive learning approaches, the random forest variant demonstrated the highest specificity (0.843 ± 0.002) and accuracy (0.832 ± 0.001), though its recall was lower at 0.585 (±0.004). Both the cost-sensitive XGBoost and LightGBM models showed similar performance, with AUC values ranging from 0.722 to 0.724. The balanced random forest, while achieving a marginally higher recall of 0.624 (±0.004), did not surpass the aforementioned models in overall performance. In contrast, the isolation forest model—used for abnormality detection—yielded the lowest performance across all evaluated metrics.

In terms of computational efficiency, the boosting models (XGBoost and LightGBM) ran the fastest. Within this group, XGBoost outperformed LightGBM regarding running time. Although the ensemble-based random forest methods (both cost-sensitive and balanced) required more computational resources due to their complexity, they still executed faster than the isolation forest model.

### Important predictors

Feature importance analysis using XGBoost with cost-sensitive learning identified prior no-show history as the most important predictor (0.128; see Table 4). Facility-related factors, such as complexity level (“Mid-sized hospital” at 0.093 and “Primary health care facility” at 0.042), were also relevant. Regional factors, including Cajamarca (0.063) and Junín (0.062), ranked among the top predictors.

**Table 4.** Feature Importance of the Best Model

Variable	Importance
No-show history (percent)	0.128
Complexity level: Mid-sized hospital	0.093
Region: Cajamarca	0.063
Region: Junín	0.062
Complexity level: Primary health care facility	0.042
Region: Puno	0.041
Assigning hour: Morning	0.037
Region: Tacna	0.036
Complexity level: Large-sized hospital	0.034
Insurance type: Ministry of Health Insurance	0.030

Additional factors included appointment timing, with “Morning” assignments having an importance of 0.037, and insurance type, with “Ministry of Health insurance” scoring 0.030. Other relevant features included regions such as Puno (0.041) and Tacna (0.036), as well as complexity level (“large-sized hospital” at 0.034).

## Discussion

### Principal results

This study evaluated the performance of multiple ML models to predict no-shows in telemedicine appointments recorded in the “Teleatiendo” system in Peru between 2019 and 2023. After processing, 1,510,297 records were included, 4.1% of which corresponded to no-shows. Among the models tested, the XGBoost model adjusted with cost-sensitive learning demonstrated the best performance, achieving a recall of 0.654 (±0.005), specificity of 0.786 (±0.002), and an AUC of 0.720 (±0.002). The most relevant predictive variables included the percentage of previous no-shows, the type of health care facility, and the patient’s geographical location. These findings underscore the importance of behavioral history and contextual factors in predicting no-shows.

Our results align with findings from other studies, albeit with variations depending on the context and the prediction techniques evaluated. In terms of performance on no-show predictions for telemedicine visits, our results are comparable to those reported in New York, where a undersampling XGBoost model achieved an AUC of 0.68 and a recall of 0.74 despite differences in population and telemedicine dynamics.^10^ While the no-show rate in Peru was 4%, considerably higher than the 2% reported in New York, the application of cost-sensitive learning strategies in our study effectively balanced the model’s performance, adapting it to the specifics of the Peruvian dataset.^16^

On the other hand, the performance metrics observed in this study were lower than those obtained in other works evaluating no-show prediction models for in-person appointments. For instance, Alshammari et al. (2020) achieved an AUC of 0.98 and a recall of 0.94 using deep neural networks on a large dataset from Saudi Arabia.^17^ This superior performance can be attributed to the availability of massive datasets, which enhance model training and mitigate overfitting risks. Similarly, recent studies highlighted the role of contextual variables such as weather conditions, socioeconomic factors, and no-show history in improving predictions for in-person appointments.^18^ Furthermore, Simsek et al. evaluated various data balancing techniques, including Synthetic Minority Over-sampling Technique (SMOTE), Random Under Sampling (RUS), and Random Over Sampling (ROS), demonstrating the importance of tailoring imbalance management strategies to the specific predictive model.^18^ This approach demonstrates how other studies could benefit from a similar methodology, avoiding reliance solely on a single technique.

Although our analysis is limited to telemedicine appointments—since the Teleatiendo system exclusively manages virtual consultations—our results can be better understood in the context of broader literature on appointment adherence. Studies in community-based clinics have consistently shown that in-person appointments tend to experience higher no-show rates compared to telemedicine. For example, Adepoju et al. found that in-person visits were more susceptible to missed appointments, likely owing to transportation barriers and scheduling complexities,^19^ while Ojinnaka et al. reported that telemedicine may reduce no-shows, especially among underserved groups, with notable differences in predictive performance between the two modalities.^20^ These findings help explain why our telemedicine model, with an AUC of 0.722, appears modest compared to some in-person models reporting AUC values as high as 0.98.^18^ The discrepancy likely reflects differences in dataset size, the breadth of contextual variables available—such as weather conditions and transportation logistics—and the intrinsic variability between virtual and in-person care. In light of these contrasts, integrating data from both telemedicine and in-person settings in future research may help clarify the determinants of no-shows and lead to improved predictive models overall.

Beyond the selection of predictive methods, models focusing on telemedicine face inherent challenges due to the nature of this care modality. Limited availability of relevant contextual data, such as weather or transportation factors, and the absence of user-specific variables like digital literacy or internet access quality restrict the predictive power of telemedicine models. Additionally, as noted by Gray et al., in-person appointments are influenced by a wider range of predictive factors, whereas telemedicine data often have narrower contextual variability, potentially explaining the observed differences in model performance.^21^ Enrichment of administrative data by real-world data extracted from electronic health records (EHR) can help improve the predictive model performance.^22,23^ Clinical decision support for no-show telemedicine appointments embedded into EHR can potentially improve access to care under limited resources and increase cost-effectiveness.^24,25^ Future studies should focus on the improvement of prediction accuracy and testing of telemedicine no-show decision support in clinical care settings.^26^

### Limitations

Our study presents some limitations that should be considered when interpreting the results. First, as a retrospective analysis based on secondary data from the “Teleatiendo” system, the findings depend on the quality and completeness of the collected data. Errors in appointment and no-show records or missing data, while excluded during preprocessing, may still have affected the analysis. However, these exclusions represented a small proportion of the total dataset, and strict selection criteria were applied to ensure the quality of the final sample.

Second, our focus was limited to teleorientation and telemonitoring appointments, which may restrict the generalizability of the findings to other telemedicine modalities, such as teleconsultations or in-person visits. Additionally, certain variables that could enhance predictions, such as digital literacy or internet access quality, were unavailable in the dataset and could not be included in the models. Nevertheless, the inclusion of variables specific to the Peruvian context, such as health care facility type and geographical location, supports the practical relevance of the results.

Despite these limitations, the study has significant strengths. It utilized a large, nationally representative dataset of over 1.5 million records, providing robust statistical power to detect behavioral patterns. Methodologically, the application of advanced ML models, such as XGBoost and LightGBM, combined with cost-sensitive learning strategies, represents an innovative approach to analyzing no-shows in telemedicine. These methodologies effectively addressed the challenges of data imbalance and demonstrated the feasibility of applying ML to optimize telemedicine systems in resource-constrained settings.

## Conclusions

This study demonstrated that ML models, particularly XGBoost with cost-sensitive learning, can moderately predict no-shows in telemedicine appointments in Peru. Key predictive factors included no-show history, type of health care facility, and geographical location, reflecting the unique dynamics of the Peruvian health care system. However, the performance of telemedicine models was lower than that observed in models for in-person appointments, which may be attributed to the absence of contextual variables such as weather or transportation and the challenges associated with telemedicine-specific factors like technological and socioeconomic barriers.

Future studies should expand the range of predictive variables to include socioeconomic factors, digital literacy levels, and internet access quality, as these could significantly enhance telemedicine predictions. Furthermore, evaluating and incorporating multiple data balancing techniques are recommended to improve model performance and avoid reliance on a single approach. Additionally, exploring other telemedicine modalities, such as teleconsultations and teleinterconsultations, could provide a broader understanding of the dynamics influencing no-shows in different care types. Finally, implementing practical strategies, such as personalized reminders and scheduling adjustments, alongside interdisciplinary collaborations, could further optimize telemedicine systems and improve health care efficiency in settings like Peru.

## Data Availability

The data sources used in this study were obtained from the Peruvian Ministry of Health and are not publicly available due to privacy and confidentiality restrictions. Interested researchers can formally request access to these data through the Ministry of Health’s transparency web platform: https://www.transparencia.gob.pe/enlaces/pte_transparencia_enlaces.aspx?id_entidad=143. All data requests are subject to review and approval by the Ministry of Health to ensure compliance with relevant regulations and data-sharing policies. The authors are not permitted to share these data directly.

## Abbreviations Used

AUC: Area Under the Curve
COVID-19: Coronavirus Disease 2019
DNN: Deep Neural Networks
EHR: Electronic Health Records
ICT: Information and Communication Technologies
ML: Machine Learning
NIH: National Institutes of Health
RUS: Random Under Sampling
ROS: Random Over Sampling
SMOTE: Synthetic Minority Over-sampling Technique
